# Progressive visceral leishmaniasis misdiagnosed as cirrhosis of the liver: a case report

**DOI:** 10.4076/1752-1947-3-7265

**Published:** 2009-06-25

**Authors:** Lydia Giannitrapani, Maurizio Soresi, Emanuele La Spada, Claudio Tripodo, Giuseppe Montalto

**Affiliations:** 1 Department of Clinical Medicine and Emerging Pathologies, University of Palermo via del Vespro 141, 90127 Palermo, Italy; 2 Department of Human Pathology, University of Palermo via del Vespro 129, 90127 Palermo, Italy

## Abstract

**Introduction:**

Visceral leishmaniasis is a potentially life-threatening infectious disease which is caused by parasites of the genus *Leishmania* and characterized in most cases by the presence of fever as well as signs and symptoms similar to those found in liver cirrhosis.

**Case presentation:**

In this case report we describe the history of a 50-year-old Caucasian man incorrectly diagnosed as having hepatitis C virus-associated liver cirrhosis, with a massive weight loss of around 100 kg during the previous 2 years. However, suspecting a lymphoproliferative disorder, we were able to make a correct diagnosis of visceral leishmaniasis by bone marrow examination. After a course of therapy with Liposomal Amphotericin-B the patient recovered and now, 20 months post-treatment, he is well and has regained a good part of the lost weight.

**Conclusions:**

This case taught us that patients with massive splenomegaly, even with a diagnosis of liver cirrhosis, should be investigated for infectious or lymphoproliferative diseases.

## Introduction

Liver cirrhosis (LC), associated with the hepatitis C virus (HCV), is very common in the Mediterranean area and is characterized by enlargement of the liver and spleen and signs of portal hypertension and pancytopenia, leading to liver failure or hepatocellular carcinoma [1]. Visceral leishmaniasis (VL) is an endemic protozoal disease of the Mediterranean area which, in its chronic course, presents signs such as liver and spleen enlargement and pancytopenia that are similar to those found in LC. Here we report the case of a patient who presented clinically with these signs, together with serologic anti-HCV positivity, who had been labeled with a diagnosis of LC which dramatically masked a picture of progressive VL.

## Case presentation

A Sicilian male patient, 50 years of age, was admitted to our ward for the first time in February 2006 following a dramatic weight loss (roughly 100 kg) and a presumptive diagnosis of liver cirrhosis related to hepatitis C virus (HCV). He was a thalassemia trait carrier and had smoked 20 to 30 cigarettes per day until 6 months previously and his past history, apart from marked familial obesity (around 150 kg), did not indicate any particular problems. However, following the appearance of asthenia and abdominal tenderness 6 years ago, laboratory analyses were performed which showed high serum levels of alanine aminotransferase (ALT). Liver cirrhosis was diagnosed on the basis of anti-HCV seropositivity (ELISA 2nd generation) with negative hepatitis B surface antigen (HBsAg) and the presence of hepato-splenomegaly associated with pancytopenia. He had no history of traveling, alcohol consumption, blood transfusion, jaundice or anything else of note and, as his HCV-RNA assay was and remains negative, he has never been treated with antiviral therapy. He was followed up for many months in a city hospital and had been receiving therapy with vitamin K and spironolactone until recently, and he has never reported having a high temperature.

**Figure 1:**
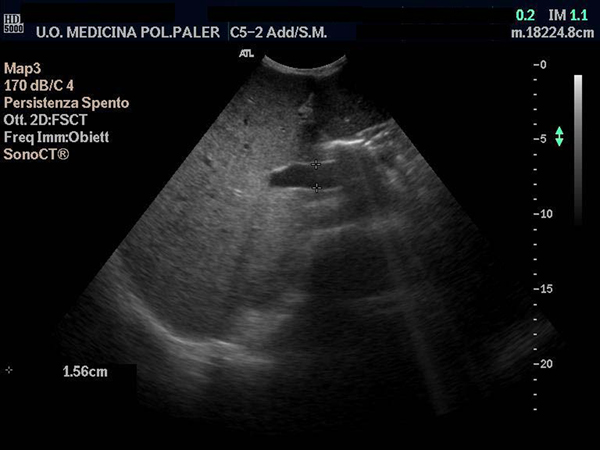
**Dilated portal vein and an increased antero-posterior diameter of the liver**.

**Figure 2:**
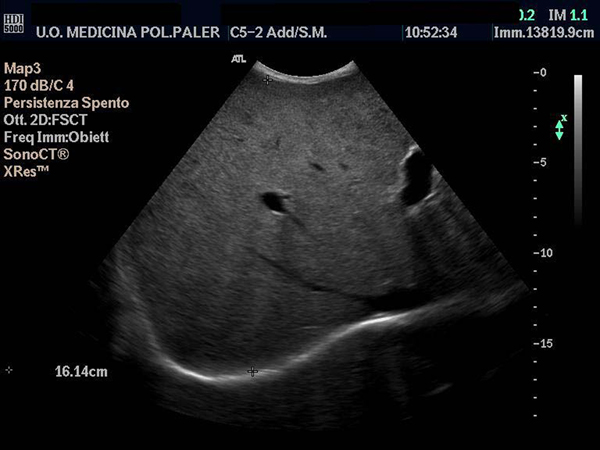
**Thin right hepatic vein and an increased antero-posterior diameter of the liver**.

He presented at our outpatient clinic with asthenia, fatigue, pedal edema, difficulty in walking, cough, hoarseness and considerable weight loss, which had started 2 years earlier. On physical examination his general condition was very poor, with loose skin folds, muscle flabbiness, dryness of the skin and mucosa, mycosis of the tongue with areas of thickening and massive hepato-splenomegaly. There was no palpable lymphadenopathy, spider nevi, palmar erythema or true gynecomastia and a total blood count performed a few days previously showed decreased hemoglobin, white blood count (WBC) and platelets. Ultrasound of the upper abdomen, performed on the same morning, confirmed massive hepatomegaly with irregular edges and a non-homogeneous hyperechoic structure, as well as thin hepatic veins with a flat Doppler waveform. The portal vein diameter was 1.4 cm (normal ≤ 1.2 cm) (Figures 1 and 2). Splenic and superior mesenteric veins were of normal caliber and showed normal response to respiration. The gallbladder and biliary tract were regular and the spleen was enlarged, with a longitudinal diameter >24 cm and a normal echo pattern. At Doppler, portal vein mean flow velocity was 37.7 cm/second, splenic artery resistance index (RI) 0.44 (nv < 0.61) and pulsatility index (PI) 0.67. There was no ascites but at the hepatoduodenal ligament there was evidence for the presence of three oval lymph nodes 2 cm in size; for all these reasons he was hospitalized.

On admission, hematologic investigations confirmed the decreased values of hemoglobin (8.8 g/dl), platelets (66.000/mmc) and WBC (1.270/mmc). Alanine amino transferase (ALT) and aspartate amino transferase (AST) were 11 and 15 IU/L; alkaline phosphatase (AP) was 266 IU/L; gamma-glutamyl transferase (γGT) 85 IU/L and total bilirubin (TB) 0.59 mg/dl. Serum total protein was 7.4 g/dl, (albumin 3.24 g/dl, γ-globulin 2.03 g/dl), total cholesterol 65 mg/dl, INR 1.17, aPTT 32.6 seconds, fibrinogen 169 mg/dl and α-fetoprotein 0.31 UI/ml. PCR for HCV-RNA was negative. Electrocardiography and chest X-ray were normal. Weight was 54.0 kg and height 165 cm.

**Figure 3:**
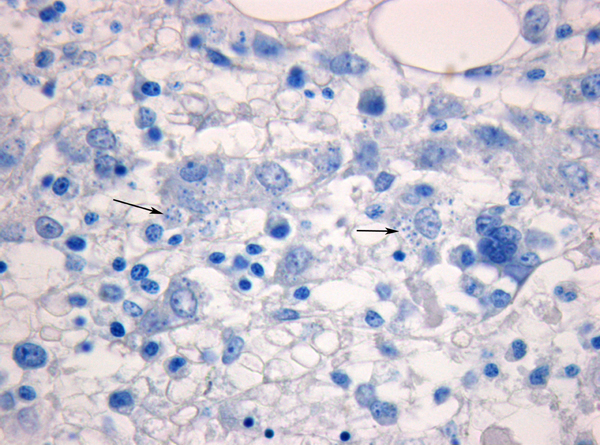
**Leishmania amastigotes inside phagocytic cells (arrows), on bone marrow biopsy, in the form of round cytoplasmic inclusions**. (Giemsa, original magnification X 400).

Because of the very marked weight loss, severe leukopenia and massive splenomegaly, which are relatively unusual in the clinical course of liver cirrhosis, and suspecting a possible lymphomatous progression of the HCV disease, we consulted a hematologist, who confirmed our suspicions and performed a bone marrow biopsy. The histologic picture showed a diffuse or nodular aggregation of histiocytic hyperplasia, containing within the cytoplasm many elements referable to the genus *Leishmania* (Figure 3). Furthermore, a serologic test for leishmania performed with indirect immunofluorescence revealed a IgG titre of 1/400. HCV-RNA, performed during hospitalization with RT-PCR was negative and serum levels of both aminotransferases were always within normal limits. To verify the presence of an immunodepressive status lymphocyte typing was performed, revealing a considerable reduction in B lymphocytes and an inversion of the CD4/CD8 ratio. HIV serology was negative. The patient therefore underwent a course of therapy with liposomal amphotericin-B (AmBisome) at a dose of 3 mg/kg per day on days 1 to 5, day 14 and day 21.

He was discharged at the end of February in relatively good general health and invited to continue therapy at home with spironolactone 100 mg/day and mycostatin half dropper four times per day for 15 days and advised a periodic outpatient check-up at our hospital.

During this time his general condition slowly but progressively improved. Liver function tests remained stable as did nutritional indexes. The patient gained weight, started to walk normally again and has now returned to almost normal activity, including a gradual return to work. Following the improvement in his clinical condition we performed a laboratory reappraisal. Oesophagogastroduodenoscopy showed hyperemic gastritis without esophageal varices. Abdominal ultrasound (US) showed hepatomegaly and splenomegaly with a longitudinal diameter of 18 cm. At Doppler the same parameters as above were confirmed. His weight was 84.5 kg, height 165 cm, BP 125/80 mmHg, PR 82/m, ALT and AST within the normal range, albumin 3.6 g/L, γ-globulin 1.56 g/dl, total cholesterol 132 mg/dl and aPTT 29 seconds. As HCV-RNA remained negative, we retested anti-HCV using a third generation method with a negative result. At this point, the initial diagnosis of liver cirrhosis, even as a disease underlying VL, seemed improbable. On the other hand, the hypothesis of a masking of a pathology that from the beginning could have been VL, albeit with some atypical tracts, is gaining significance. The patient is at present being followed-up and is on support therapy, which includes psychological support and alimentary education training following a diet with oligo elements and vitamin supplements under the supervision of a nutritionist.

## Discussion

Leishmaniasis is a parasitic disease transmitted by the bite of sand flies. Three main forms are known: cutaneous, mucocutaneous and visceral. VL presents a sub-acute or chronic course and if not treated with a specific therapy the disease almost invariably leads to terminal cachexia and death [2]. The case we observed is interesting because a number of considerations can be made. First of all, the presence of anti-HCV positivity, together with hypertransaminasemia and liver and spleen enlargement, led to a diagnosis of HCV-related liver cirrhosis but the negative viremia associated with pancytopenia ruled out the need for a cycle of antiviral therapy.

The possibility of mistaking a chronic liver disease for VL has been recently reported in the literature [3]. Prakash et al presented the case of a patient with clinical and biochemical features of liver cirrhosis in whom a correct diagnosis of VL was made after liver biopsy, which demonstrated the parasite in the Kupffer cells. Unfortunately, the patient died after commencing therapy with sodium antimony stibogluconate [3]. Acute hepatitis has also been reported as a presenting manifestation of VL [4]. In our case, another feature which delayed the correct diagnosis was the constant absence of fever in spite of the lengthy duration of the illness, although it is well known that VL is usually associated with fever. This event, although rare, has also been reported by other authors [5], but a lack of this fundamental symptom can obviously delay or divert from a correct diagnosis. Another component that most likely contributed to the misdiagnosis was weight loss. Since the patient was severely obese, his loss in weight, at least initially, was seen as a positive factor for his health, but obviously not when this went beyond normal limits.

Moreover a certain carelessness on the part of the patient about his condition and the presence of the anti-HCV positivity, contributed to delaying the true diagnosis. Even in our clinic the diagnosis of VL was made by chance, because our initial suspicion was a possible lympho-proliferative disorder involving the liver and spleen, which were too large in volume for a common HCV-associated LC. This orientation was based on a recent case of ours of lymphomatous liver in a HCV-positive patient, which had luckily been identified [6]. Therefore, we were surprised to identify leishmania in the bone marrow and the diagnosis was confirmed both with serology and a successful course of specific therapy. Naturally, some of the data obtained were not in agreement with a diagnosis of liver cirrhosis, including the lack of portal hypertension at ultrasound using color-Doppler, as well as the lack of oesophageal varices at endoscopy. We consequently investigated for the presence of an underlying HCV-associated chronic liver disease (CLD). HCV-RNA assay, repeated during the course of the disease, was always negative and the re-evaluation of anti-HCV positivity, using a third generation assay (ELISA 3), was also negative, so there was probably no underlying CLD. Indeed, the anti-HCV positivity, although it was performed using a second generation assay, may be seen as a false positive result due to hypergamma globulinemia or, more specifically, linked to the production of IgM with rheumatoid factor (RF) characteristics. In fact, it has been reported that an increase in IgM-RF production is an autoimmune characteristic of VL [7] and it is well known that the presence of RF could interfere with correct anti-HCV detection, causing significant false positive reactivity [8]. Unfortunately, we did not perform RF before treatment and now RF is negative, so our suggestion remains speculative. Another possibility is that some antigens of leishmania may have a cross reaction with HCV, but this hypothesis needs further investigation. Confirmation of a possible chronic liver disease should be performed by liver biopsy, but at the moment we consider it unethical and unnecessary to perform an invasive investigation, both because the patient is unwilling and there are also no indications for alternative treatment.

## Conclusions

Here we report the case of a patient with massive weight loss, severe hepato- and splenomegaly, pancytopenia and anti-HCV positivity, who had been labeled as cirrhotic. The diagnosis of VL, made by chance, allowed us to give the patient specific treatment, without which he would most probably have died. This experience should induce physicians to further investigate and, if necessary, re-evaluate a given diagnosis (in this specific case liver cirrhosis, whether it is of viral or non-viral origin), when its course or clinical signs deviate from the current standard diagnostic criteria.

## Abbreviations

ALT: alanine amino transferase; AP: alkaline phosphatase; aPTT: activated partial thromboplastin time; AST: aspartic amino transferase; BP: blood pressure; CLD: chronic liver diseases; γGT: gamma glutamyl transpeptidase; HBsAg: hepatitis B surface antigen; HCV: hepatitis C virus; HIV: human immunodeficiency virus; IgG: immunoglobulin G; IgM: immunoglobulin M; INR: international normalized ratio; LC: liver cirrhosis; PCR: polymerase chain reaction; PR: pulse rate; RBC: red blood cells; RF: rheumatoid factor; TB: total bilirubin; US: ultrasound; VL: visceral leishmaniasis; WBC: white blood cells.

## Consent

Written informed consent was obtained from the patient for publication of this case report and accompanying images. A copy of the written consent is available for review by the Editor-in-Chief of this journal.

## Competing interests

The authors declare that they have no competing interests.

## Authors' contributions

LG wrote the manuscript and participated in the literature review, MS participated in the clinical management of the patient, ELS was the attending physician who conducted the clinical management of the patient, CT was the pathologist who performed the bone marrow biopsy and histologic examination and participated in the literature review and GM participated in the literature review, collection and analysis of pertinent information and was a contributor in writing the manuscript.
